# Toxicity assessment of hexafluoropropylene oxide-dimer acid on morphology, heart physiology, and gene expression during zebrafish (*Danio rerio*) development

**DOI:** 10.1007/s11356-022-24542-z

**Published:** 2022-12-03

**Authors:** Sylvia Gong, Flannery McLamb, Damian Shea, Jeanne P. Vu, Miguel F. Vasquez, Zuying Feng, Kesten Bozinovic, Ken K. Hirata, Richard M. Gersberg, Goran Bozinovic

**Affiliations:** 1 Boz Life Science Research and Teaching Institute, San Diego, CA USA; 2 Division of Extended Studies, University of California San Diego, La Jolla, CA 92093-0355 USA; 3 School of Public Health, San Diego State University, San Diego, CA USA; 4 Statera Environmental, Raleigh, NC USA; 5 Graduate School of Arts and Sciences, Georgetown University, Washington, DC USA; 6 Division of Biological Sciences, University of California San Diego, La Jolla, CA 92093-0355 USA

**Keywords:** Embryology, *Danio rerio*, Gene expression, Toxicology, HFPO-DA, RNA sequencing

## Abstract

Hexafluoropropylene oxide-dimer acid (HFPO-DA) is one of the emerging replacements for the “forever” carcinogenic and toxic long-chain PFAS. HFPO-DA is a polymerization aid used for manufacturing fluoropolymers, whose global distribution and undetermined toxic properties are a concern regarding human and ecological health. To assess embryotoxic potential, zebrafish embryos were exposed to HFPO-DA at concentrations of 0.5–20,000 mg/L at 24-, 48-, and 72-h post-fertilization (hpf). Heart rate increased significantly in embryos exposed to 2 mg/L and 10 mg/L HFPO-DA across all time points. Spinal deformities and edema phenotypes were evident among embryos exposed to 1000–16,000 mg/L HFPO-DA at 72 hpf. A median lethal concentration (LC_50_) was derived as 7651 mg/L at 72 hpf. Shallow RNA sequencing analysis of 9465 transcripts identified 38 consistently differentially expressed genes at 0.5 mg/L, 1 mg/L, 2 mg/L, and 10 mg/L HFPO-DA exposures. Notably, seven downregulated genes were associated with visual response, and seven upregulated genes were expressed in or regulated the cardiovascular system. This study identifies biological targets and molecular pathways affected during animal development by an emerging, potentially problematic, and ubiquitous industrial chemical.

**Supplementary Information:**

The online version contains supplementary material available at 10.1007/s11356-022-24542-z.

## Introduction

Per- and polyfluoroalkyl substances (PFAS) represent a diverse group of over 4700 chemicals that contain carbon–fluorine bonds, the strongest chemical bond in organic chemistry. PFAS (Supplementary Table S1) are widely used in many industrial and consumer products including waterproof and non-stick items, such as pans, food wrappers, waterproof fabrics, lubricants, surfactants, chemically inert composites, insulators, firefighting foams, and plastics (Beekman et al. 2016; US EPA 2022b; L. H. Yang et al. 2022). The strength of the carbon–fluorine bond makes most PFAS resistant to degradation and persistent in the environment. PFAS exposures have been associated with adverse health effects in animals, including cancer (Barry et al. 2013; Biege et al. 2001; Shearer et al. 2021; Vieira et al. 2013), endocrine disruption (Dhillon et al. 2019; Du et al. 2013), hepatotoxicity (K. Li et al. 2017), immunotoxicity (Dewitt et al. 2015), and developmental and reproductive toxicity (Conley et al. 2019; Salimi et al. 2019). The ubiquitous environmental presence of PFAS prompted the implementation of the Environmental Protection Agency PFOA Stewardship Program in 2006, requiring major fluoropolymer manufacturers to reduce or eliminate emissions of perfluorooctanoic acid (PFOA, C_8_HF_15_O_2_) and related chemicals (US EPA 2006). In 2012, PFAS were detected in over 97% of screened human serum samples (Lewis et al. 2015; US CDC 2012). The primary route of human exposure is through water (Franke et al. 2021); in 2016, drinking water supplies for six million Americans exceeded the United States Environmental Protection Agency’s (US EPA) PFOS and PFOA lifetime health advisory level of 70 ng/L, which has been updated to 0.02 ng/L and 0.004 ng/L, respectively (Hu et al. 2016; US EPA 2022c).

Since increasing PFAS chain length is associated with biological and chemical stability and toxicity (Hagenaars et al. 2011; Jantzen et al. 2016), long-chain PFOA was succeeded by shorter-chain, potentially less toxic, and less bioaccumulative variants. Hexafluoropropylene oxide-dimer acid (HFPO-DA, C_6_HF_11_O_3_), commonly referred to as GenX, is a short-chain polymer processing aid used as an alternative to PFOA to make fluoropolymers (Brandsma et al. 2019; US EPA 2022a). Having six-carbon atoms and an ether group (Supplementary Table S1) is supposedly less toxic than the eight-carbon PFOA; it is environmentally persistent (Beekman et al. 2016), highly stable, and water-soluble (Hassell et al. 2020; Liberatore et al. 2020), with a half-life of 20 h in mice (Gannon et al. 2016) compared to approximately 1000 h for PFOS (Chang et al. 2012). US EPA derived a final lifetime health advisory level in drinking water as 10 ng/L (US EPA 2022c). Cape Fear River, NC, US, a drinking water source, was reportedly contaminated by a nearby manufacturer and contained up to 4500 ng/L HFPO-DA in 2016 (Sun et al. 2016). HFPO-DA was the largest proportion of PFAS in all surface water samples along the German coastline at a mean concentration of 1.6 ng/L in 2017 (Joerss et al. 2019) and was detected in 90% of surface water samples at a mean concentration of 30 pg/L in the Arctic Ocean and its neighboring waterbodies in 2018 (Joerss et al. 2020). In 2020, rain and well-water assays in Ohio and Indiana detected HFPO-DA in six sites at 0.2–5 ng/L (Galloway et al. 2020). Evidence for HFPO-DA hepatotoxicity (Conley et al. 2021; Shi et al. 2019), and metabolic (Conley et al. 2019) and endocrine disruption (Xin et al. 2019), the risk of both acute and chronic environmental exposures, and a lack of human-health exposure data, necessitate comprehensive toxicological and ecological risk assessment.

Gene expression changes during organogenesis, fetal, and infantile development due to chemical exposures can increase susceptibility to disease later in life (Grandjean et al. 2008; Pennings et al. 2016; Peterson et al. 2000), demonstrating how environmental exposure to HFPO-DA may affect embryogenesis. *Danio rerio* (zebrafish) is a well-established human disease, genetics, embryology, and physiology model organism (Lieschke and Currie 2007), whose genome (Howe et al. 2013) has at least one ortholog of 71.4% of human genes and 82% of human disease-related genes. Its sensitivity to chemicals (von Hellfeld et al. 2020), compatibility with mammalian system toxicity assessment (Ducharme et al. 2015), and well-studied development (Kimmel et al. 1995) allow for robust assessments of toxicological developmental phenotypes. The goal of our study is to characterize the in vivo toxicity potential of HFPO-DA by evaluating adverse physiological effects at acute exposure levels and determining molecular targets of HFPO-DA exposures. We identified adverse acute exposure effects on morphological and physiological phenotypes, and gene expression alterations via shallow RNA sequencing during zebrafish early development.

## Materials and methods

### Zebrafish maintenance

Adult zebrafish AB strain (Carolina Biological; Burlington, NC, USA) were acclimated to lab conditions for six months and maintained per established laboratory guidelines (Institute of Laboratory Animal Resources (US). Committee on Care and Use of Laboratory Animals, 1986). Ten zebrafish per liter were housed in a recirculating system in 1.3- or 3.3-L polycarbonate tanks maintained at 28.5 ± 1 °C on a 14 h light:10 h dark cycle. Adults were fed Tetramin® Tropical Flake Food (Tetra, Blacksburg, VA, USA) and brine shrimp (Brine Shrimp Direct, Ogden, UT, USA) daily. Experimental procedures, including non-surgical tissue sampling and fish embryo culturing and maintenance, were approved by the San Diego State University Institutional Animal Care and Use Committee (Animal Welfare Assurance Number A3728-01).

### Embryo collection

Breeding tanks with one female and two male F1 generation zebrafish were established 24 h before embryo collection. Adults were bred for 1 h after the onset of the light cycle, and embryos were collected in Pyrex glass dishes covered with mesh; embryos were staged (Kimmel et al. 1995) under a stereo microscope (AmScope Compact Multi-Lens Stereo Microscope, model #SE306R-A) at 30 × magnification. Debris and abnormally developing embryos manifesting severe cleavage asymmetry, detached cytoplasm, and/or broken chorions were removed. The remaining embryos were kept at 28.5 °C (Precision Microbiological Incubator, Thermo Scientific, Waltham, MA, USA) in deionized water reconstituted with Instant Ocean® (Instant Ocean Spectrum Brands, Blacksburg, VA, USA) to 60 μg/L, adjusted to pH 6.6–7.6.

### Chemicals and exposure media preparation for embryo exposure

Due to the high solubility of HFPO-DA, reportedly over 751 g/L (US EPA 2021b; Nixon and Lezotte 2008), exposure solutions were prepared by directly adding HFPO-DA (Undecafluoro-2-methyl-3-oxahexanoic acid, CASRN: 13,252–13-6; 97% purity, Catalog No. 2121–3-13, SynQuest; Alachua, FL, USA) to embryo water without using a vehicle. Because pH affects embryo mortality (Andrade et al. 2017), assays using pH-neutralized HFPO-DA (pH = 6.6–7.6) were performed. pH neutralization was performed by titrating with 0.765 M NaOH and 6 M HCl (Cassar et al. 2019; OECD 2020; S. Y. Williams and Renquist 2016) and checking with a pH meter (PH220-C, Extech Instruments, Nashua, NH, USA). Prepared exposure solutions were stored in glass bottles at 4 °C one day prior to exposure. Individual embryos in 24-well plates were exposed to 2 ml of 0.5 mg/L, 1 mg/L, 2 mg/L, 10 mg/L, 1000 mg/L, 4000 mg/L, 6000 mg/L, 8000 mg/L, 10,000 mg/L, 12,000 mg/L, 16,000 mg/L, and 20,000 mg/L titrated HFPO-DA from 3 to 4 hpf, the sphere developmental stage (Kimmel et al. 1995), until 72 hpf. Negative control embryos were grown in embryo water, and positive control embryos were exposed to 4 mg/L 3,4-dichloroaniline. Embryos were incubated at 28.5 °C (Precision Microbiological Incubator, Thermo Scientific, Waltham, MA, USA) throughout exposures. Fifty percent of the exposure solution in each well was refreshed daily. pH and dissolved oxygen (DO) were noted for each plate daily. Exposures were deemed valid if over 80% of negative control embryos survived and developed normally, while over 30% of positive control embryos died (OECD 2013). Exposure sample sizes per treatment are listed in Supplementary Table S2.

### Mortality, heart rates, and morphology

Embryo mortality was assessed at 24 hpf, 48 hpf, and 72 hpf using an inverted microscope (Research Grade Inverted Microscope, Infinite Optical System, Fisher Scientific, catalog # 03–000-013) and defined by coagulation of the embryo at 24 hpf or lack of heartbeat at 48 hpf and 72 hpf. Median lethal concentrations (LC_50_) were calculated using a sigmoidal 4PL curve via Prism (Version 9, GraphPad Software, San Diego, CA, USA).

At least 50% of embryos from each exposure were randomly selected to assess heart beats per minute (HBPM) at 24 hpf, 48 hpf, and 72 hpf. Embryo plates were acclimated to room temperature for 10 min, and each embryo was placed under the microscope light for 30 s before heartbeats were counted for 30 s. Embryos at 72 hpf were then pooled into batches of 8–10 and snap-frozen and stored at − 80 °C until RNA extraction.

Surviving larvae were imaged within wells or depression slides (SeBaCam, Laxco); lateral view images (40 ×) were used to identify malformations (von Hellfeld et al. 2020). Pericardial edema was categorized as swelling peripheral to the pericardium, and yolk-sac edema was characterized as sections of the yolk-sac that were transparent rather than pigmented and separated from the yolk-sac. Spinal deformations were qualified as curved if a line traced from eye to tip of tail deviated from 180° during any 1 mm segment aside from the head-trunk angle, or if the head-trunk angle was greater than 60°. These malformations were further categorized as kyphosis (excessive forward curve), lordosis (excessive inward curve), or scoliosis (sideways curve). The severity of malformation at 72 hpf was scored on a scale of 1 to 4, modified from the existing scoring method (Gaballah et al. 2020): Normally developing embryos were scored as 1, single malformation as 2, two malformations as 3, and four or greater malformations as 4. Effect concentration (EC_50_) was calculated using a sigmoidal 4PL fit curve.

### Bioconcentration of HFPO-DA from water

To assess the bioaccumulative potential of HFPO-DA in zebrafish embryos, embryos were exposed to 4000 mg/L HFPO-DA at 28.5 °C until 72 hpf with 50% water changes at 24 h and 48 h. Embryos were snap-frozen at − 80 °C and collected into 15 mL high-density polypropylene Falcon tubes spun down at 4500 rpm for 2 min; the excess liquid was removed, and embryo samples were re-stored at − 80 °C. HFPO-DA embryo body burden analysis was performed by Statera Environmental, Inc. (Raleigh, NC, USA). Pooled embryos were collected, homogenized, and dried (total dry weight: 0.702 g, 2978 embryos in 0 mg/L; 1.026 g, 3067 embryos in 4000 mg/L), then subsampled to 3 replicates per condition. A bioconcentration factor (BCF) was calculated using the following equation (Veith et al. 2011):

$$BCF=\frac{{C}_{F}}{{C}_{w}}$$

where *C*_*F*_ is the average concentration of HFPO-DA in embryos, and *C*_*W*_ is the average concentration of HFPO-DA in water at 0 h, 24 h, 48 h, and 72 h. Analysis of HFPO-DA in water and embryo samples followed modified methods (Gaballah et al. 2020; US EPA 2021a)*.* Native and mass-labeled standards (Wellington Laboratories, Inc., ON, CA) were diluted in 95% methanol:5% aqueous 2.5 M NaOH. All standards, samples, and extracts were stored in plastic at 4 °C. Water samples were diluted in 5% acetonitrile:95% water, fortified with perfluorononanoic acid (PFNA) as a surrogate and ^13^C_3_-HFPO-DA as the internal standard, and directly injected. Embryo samples were extracted by protein precipitation, pooled and subsampled to obtain three 20.0 mg replicates (~ 100 embryos), flash frozen, and homogenized using a Bead Ruptor 12 (Omni International, GA, USA) with ∼250 mg of zirconia/silica beads (1.0-mm dia) in 500 µL 0.1 M formic acid that was fortified with PFNA. The protein was precipitated with 500 µL acetonitrile fortified with the internal standard and centrifuged at 15,000 rpm for 15 min at 4 °C. A 50-µL aliquot of the extract was diluted with 200 µL of aqueous 0.4 mM ammonium formate.

Instrumental analysis of standards, water, and embryo extracts was performed on an Agilent Infinity II UHPLC (Santa Clara, CA, USA) outfitted with an Agilent PFC-Free HPLC Conversion Kit. Separation was performed on an Agilent Poroshell 120 EC-C18 column and guard column, with a Restek Ultra Aqueous C18 trap column (Bellefonte, PA, USA) and Agilent Zorbax Eclipse Plus C18 delay column, under the following conditions: mobile phase A (2 mM ammonium acetate in 5:95 acetonitrile:water), mobile phase B (acetonitrile), needle/seat wash (50:50 acetonitrile:water) using two cycles of the seat back flush and needle wash (10 s each), seal wash (10:90 isopropanol:water), injection volume 5.00 µL, flow rate 0.50 mL min^−1^, and solvent gradient mobile phase A; mobile phase B being initial (85%:15%), 1.00 min (85%:15%), 5.00 min (10%:90%), 6.40 min (10%:90%), and 6.50 min (85%:15%), end time 9.50 min, and column temperature 50.0 °C. The UHPLC was coupled to an Agilent 6495C triple quadrupole mass spectrometer (MS) operated in ESI negative mode, MRM scan, gas temperature 150 °C, gas flow 8L min^−1^ nebulizer 45 psi, sheath gas heater 200 °C, sheath gas flow 8 L min^−1^ capillary 3500 V, and nozzle voltage 0 V. MRM transition was precursor ion 284.9, quantification product ion 168.9, and confirmation ion 184.9 with ion ratio of 1.95 ± 0.20. The quantification reference compound was ^13^C_3_-HFPO-DA.

The stability of HFPO-DA concentration in water was assessed by preparing media following embryo exposures, with the exclusion of embryos. Exposure media (10 mL) was aged at 28.5 °C in an incubator and collected at 0 h, 24 h, 48 h, and 72 h with 50% water changes at 24 h and 48 h.

### RNA isolation, cDNA library preparation, and shallow RNA sequencing

Total RNA was isolated from pooled samples of 8–10 embryos with four pools for each exposure using the TRIzol reagent protocol (Invitrogen, Carlsbad, CA, USA). Briefly, TRIzol was added to frozen embryos, before allowing embryos to thaw. Samples were homogenized in a Bead Mill 4 (Fisher Scientific, PA) using sterile 2 mm glass beads and resuspended in 50 µL RNase-free water. The quantity and quality of samples were determined using a NanoDrop 2000 (A260/280 and A260/230 > 1.8). Between 500 and 750 ng of total RNA per sample were delivered to the Scripps Research Genomics Core (San Diego, CA, USA) for library prep and shallow RNA sequencing (RNA-Seq).

Sample quality was assessed using a 2100 Bioanalyzer 2100 (Agilent Technologies) (RIN > 8.5). Library preparation was performed using the HTP RNA-Seq Library Prep Kit (iGenomX Inc, San Francisco, CA, USA). Briefly, barcoded oligo dT primers were added to 50 ng RNA per sample and reverse transcribed. Sample cDNA products were combined, cleaned, and run through PCR with 0.5 µM barcoded PCR primers (p5 and p7 sequences, Illumina, San Diego, CA, USA). Purified PCR products were sequenced using a NextSeq2000 sequencer (paired-end mode; read1: 26 bp, read2: 94 bp), generating greater than 1.5 million paired-end reads for each sample.

### Shallow RNA sequencing quality control and data processing

Sample data were demultiplexed by Scripps Research Genomics Core using BBTools (version 37.62) (Bushnell et al. 2017). Raw shallow RNA-Seq data quality was assessed using FastQCR (Kassambara 2019), and UMIs were extracted using UMI-tools (version 1.1.2) (Smith et al. 2017). To remove adapters and optimize for differential expression detection, Trimmomatic (version 0.40) was used to remove the first 16 bases of each read, trim reads according to a sliding window of length 4 and minimum Phred score of 20, and to remove resulting reads shorter than 20 bases (Bolger et al. 2014). SortmeRNA (version 2.1) was used to filter out contaminating rRNA (Kopylova et al. 2012). The remaining reads were aligned to zebrafish genome assembly version 10 (GRCz10) using STAR (version 2.7.3a) (Dobin et al. 2013). PCR deduplication was performed using extracted UMIs via UMI-tools (Smith et al. 2017). Read counts per gene were quantified using HTSeq (version 0.13.5) with default settings (Putri et al. 2022), and transcript variants were treated as single genes. Gene expression was normalized using a trimmed mean of M values (TMM) (Robinson & Oshlack 2010). Differential expression analysis was performed in parallel using the noiseqbio function of NOISeq (version 2.14.1) on data filtered in increments of 0.25 from 0 to 5 CPM (Tarazona et al. 2015). gGenes of > 2.75 average CPM were selected for further analysis. Raw and processed shallow RNA-Seq data were deposited in the National Center for Biotechnology Information Gene Expression Omnibus under the accession GSE198976 (https://www.ncbi.nlm.nih.gov/geo/query/acc.cgi?acc=GSE198976).

Shallow RNA-Seq results were verified via qRT-PCR analysis, using statistically significant six upregulated (minimum fold change of 2.59-fold) and five downregulated (minimum fold change of 1.56-fold) genes in all exposures. *actb1* was selected for housekeeping gene normalization based on its reliability (McCurley et al. 2008; Tang et al. 2007; Xu et al. 2016) and stable expression within and between exposures. Primers were designed using Primer-BLAST (J. Ye et al. 2012) with standard parameters (Supplementary Table S3). Equal concentrations from three shallow RNA-Seq replicates were pooled for qRT-PCR per iTaq Universal SYBR Green One-Step kit protocol (Bio-Rad, Hercules, CA, USA)*.* qRT-PCR results were analyzed with the 2^−ΔΔ*Ct*^ method. To compare expression values between qRT-PCR and shallow RNA-Seq, counts from shallow RNA-Seq were normalized using the transcript per million (TPM) (B. Li and Dewey 2011) and TMM methods relative to *actb1*.

### Statistical analysis

Data normality for embryo survival, morphology, and HBPM was evaluated using Shapiro–Wilk (*p* < 0.05), and equal variance was tested using the Brown-Forsythe method (*p* < 0.05). Survival, HBPM, and morphometric measurements between exposure groups were analyzed by one-way ANOVA with Tukey’s HSD if data were normal and equally variable. Nonparametric data were analyzed with a Kruskal–Wallis ANOVA with Dunn’s multiple comparisons for equal variances, or Steel–Dwass’ multiple comparisons for unequal variance. Dose–response curves for malformations were fitted to the model with the lowest Akaike Information Criterion, *p* < 0.05. JMP Pro (version 14.0, SAS Institute Inc, Cary, NC, USA) was used for statistical analysis and figure generation.

Pairwise differential gene expression was determined using the noiseqbio function of NOISeq (version 2.14.1), with the thresholds *q* > 0.9 and FDR < 0.1 (Tarazona et al. 2015). Agglomerative hierarchical clustering was performed using Ward’s method in the R package cluster (Maechler et al. 2022). Enrichment in Gene Ontology (GO, Open Biological Ontologies Foundation) biological process terms was quantified via the Cytoscape plugin BiNGO (version 3.0.5) using total zebrafish genome annotation as background (hypergeometric test, Benjamini–Hochberg *p* < 0.05) (Maere et al. 2005). Figures were generated using R (version 4.1.2) packages ComplexHeatmap (Gu et al. 2016) and GOplot (Walter et al. 2015).

## Results

### *Embryo* survival and morphology

**Fig. 1:**
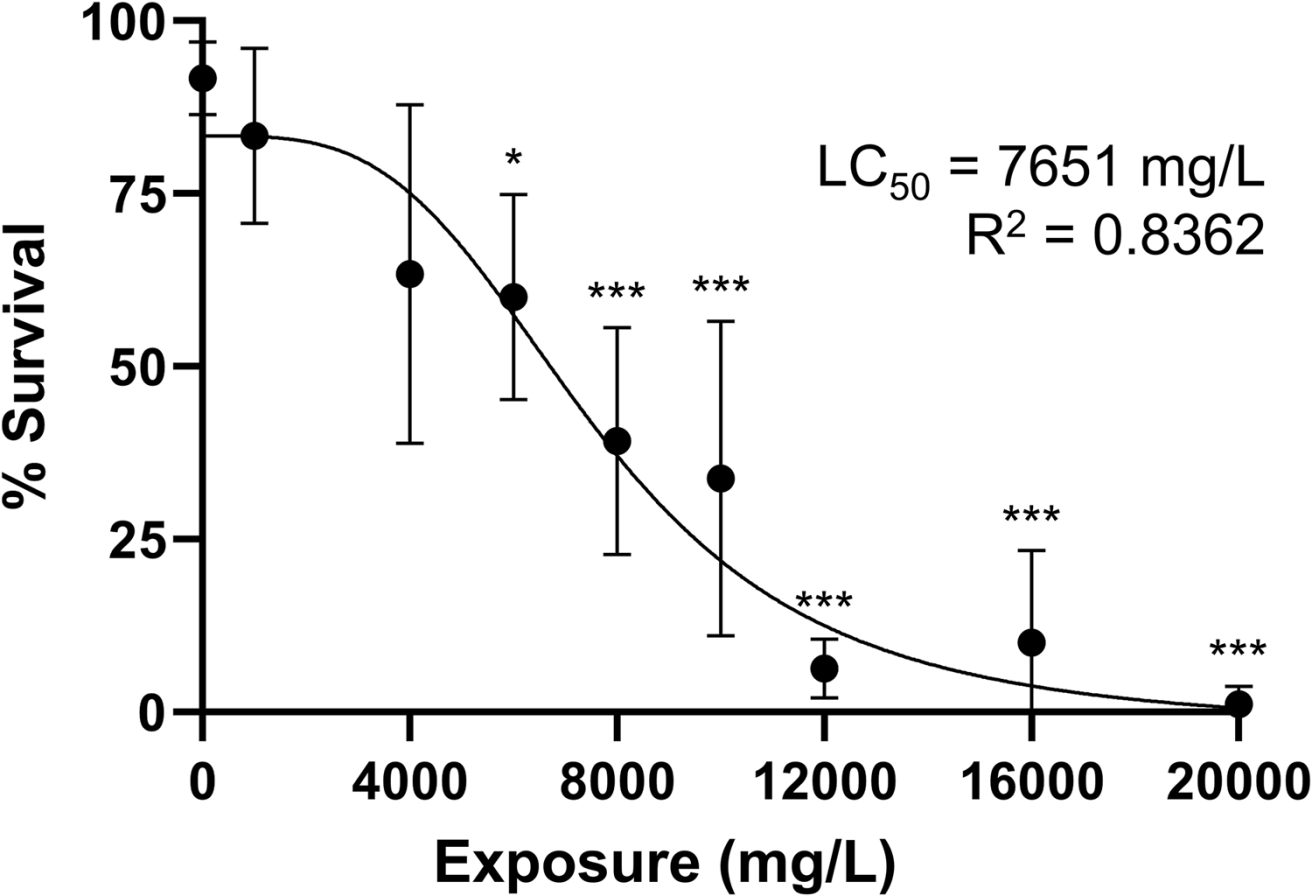
Concentration–response curve at 72 hpf for zebrafish embryo HFPO-DA exposure. Each point represents the mean percent survival following 14, 6, 6, 6, 13, 8, 8, 8, and 9 trials (10 embryos per trial) in HFPO-DA exposures of 0 mg/L, 1000 mg/L, 4000 mg/L, 6000 mg/L, 8000 mg/L, 10,000 mg/L, 12,000 mg/L, 16,000 mg/L, and 20,000 mg/L, respectively. LC_50_ is 7651 mg/L (95% CI = 5976–8080 mg/L). Asterisks indicate significant differences from the control (Kruskal–Wallis ANOVA (*p* < 0.001) with a Dunn’s multiple comparisons test (*p* < 0.05*, *p* < 0.001***). Error bars represent standard deviation. The curve was fitted using sigmoidal 4PL

Untitrated exposure survival at concentrations of 60–150 mg/L HFPO-DA was significantly lower than control (*p* < 0.05; Supplementary Fig. S1 and Table S4), with an LC_50_ of 51.12 mg/L. Titrated exposure survival was significantly lower than control from 6000 to 20,000 mg/L (*p* < 0.05), with an LC_50_ of 7651 mg/L (95% CI 5976–8080 mg/L; Fig. 1, Supplementary Table S2).

**Fig. 2:**
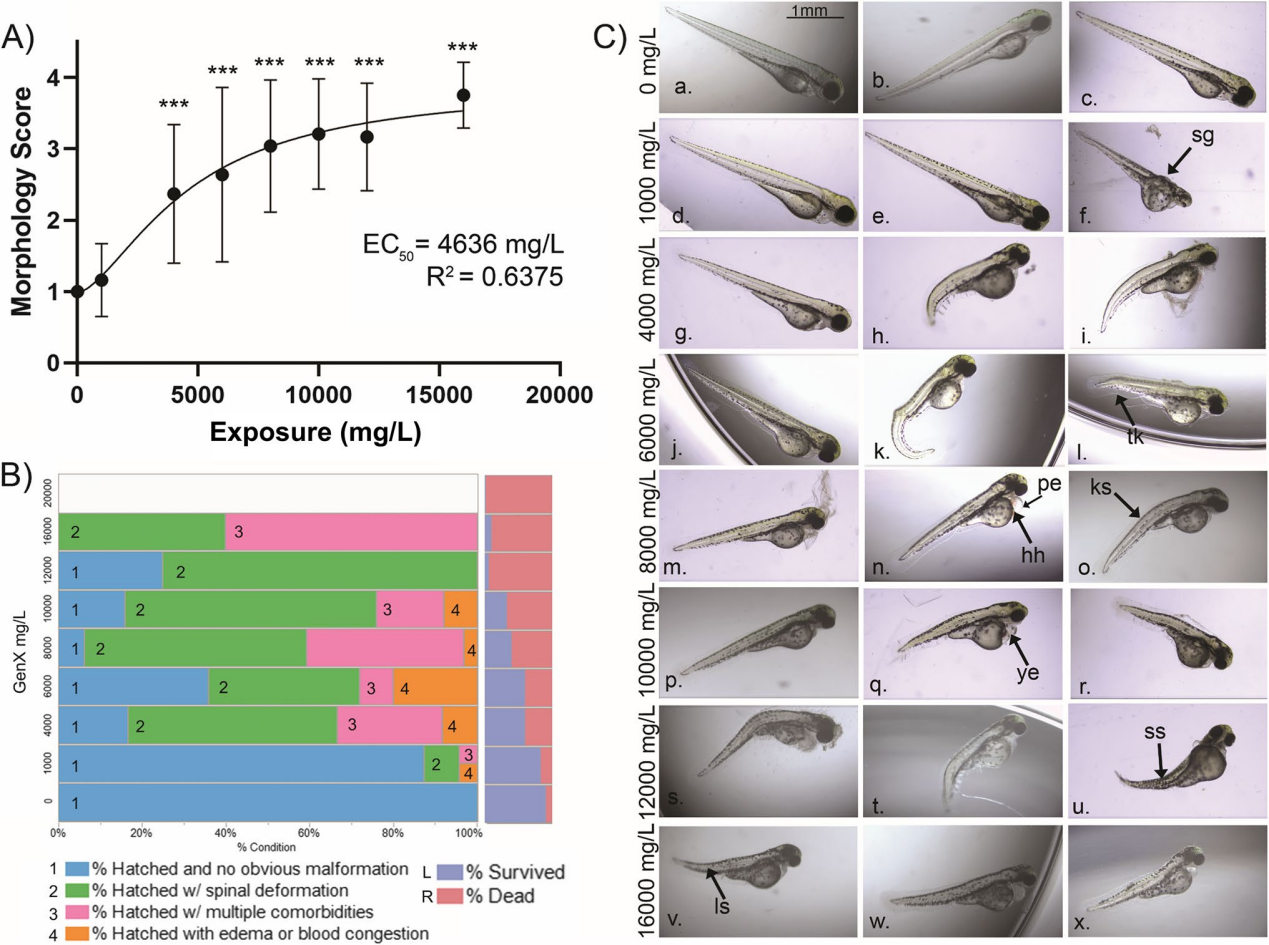
Morphological assessment of zebrafish embryos exposed to high levels of HFPO-DA. Embryos were exposed to 0 mg/L, 1000 mg/L, 4000 mg/L, 6000 mg/L, 8000 mg/L, 10,000 mg/L, 12,000 mg/L, and 16,000 mg/L from 3 to 72 hpf. Only surviving embryos are included (Supplementary Table S2). (**A**) Morphology scores (1:4, with a score of 1 indicating no malformations, 2-mild, 3-moderate, and 4-severe). EC_50_ of malformation is 4636 mg/L (95% CI = 3207–11,179 mg/L). Error bars represent standard deviation. The curve was fitted using sigmoidal 4PL. Asterisks indicate significant differences from the control (Kruskal–Wallis ANOVA (*p* < 0.001) with a Dunn’s multiple comparisons test; *p* < 0.001***). (**B**) Incidences of spinal deformation, edema/blood congestion, or multiple comorbidities (left); percent surviving and dead embryos at 72 hpf (right). (**C**) Images of exposed embryos. Arrows indicate example malformations, denoted by sg = stunted growth (2C.f), tk = tail kink (2C.k), hh = hemorrhaging (2C.n), pe = pericardial edema (2C.n), ks = kyphosis (2C.o), ye = yolk-sac edema (2C.q), ss = scoliosis (2C.u), ls = lordosis (2C.v)

Malformations identified in the 1000–16,000 mg/L exposures range increased in severity with increasing exposure concentration (Fig. 2A, Supplementary Table S2), with the lowest observed adverse effect level (LOAEL) at 4000 mg/L (*p* < 0.001). The half-maximal effective concentration (EC_50_) was 4636 mg/L (95% CI = 3207–11,179 mg/L). Stunted growth (Fig. 2C.f, 1000–16,000 mg/L) was marked by spinal deformations, including kyphosis (Fig. 2C.o, 4000–16,000 mg/L), scoliosis (Fig. 2C.u, 4000–16,000 mg/L), lordosis (Fig. 2C.v, 4000–16,000 mg/L), and tail kinks (Fig. 2C.k, 4000–16,000 mg/L). Other malformation phenotypes were pericardial edema and hemorrhage (Fig. 2C.n, 1000–10,000 mg/L) and yolk-sac edema (Fig. 2C.q, 1000–10,000 mg/L). Forty-six percent of embryos exposed to 1000–16,000 mg/L HFPO-DA manifested spinal deformations, while edemas/hemorrhaging were apparent in 21.2% of embryos.

### Embryo heart rate

**Fig. 3:**
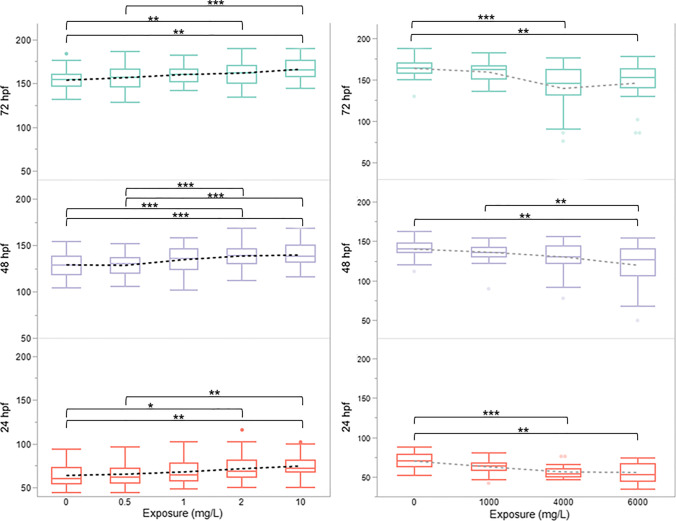
Zebrafish embryo HBPM increases with HFPO-DA exposure. Embryos were exposed to (**A**) 0 mg/L, 0.5 mg/L, 1 mg/L, 2 mg/L, 10 mg/L HFPO-DA (*n* = 54, 53, 54, 56, 44 embryos measured, respectively) or (B) 0 mg/L, 1000 mg/L, 4000 mg/L, 6000 mg/L HFPO-DA (*n* = 29, 28, 24, 22, respectively). HBPM were recorded at 24 hpf, 48 hpf, and 72 hpf. Data for 24 hpf were not normally distributed and were analyzed using Kruskal–Wallis ANOVA with a Steel–Dwass multiple comparisons test. Statistical analysis for 48 hpf and 72 hpf were performed using one-way ANOVA (*p* < 0.001) with Tukey’s HSD (*p* < 0.001***, *p* < 0.01**). Error bars represent the standard deviation

Embryo HBPM at 72 hpf was significantly higher relative to control at the LOAEL of 2 mg/L (NO observed adverse effect level, NOAEL = 1 mg/L) and at 10 mg/L (Fig. 3, *p* < 0.01; Supplementary Table S5). HBPM increased during all exposure concentrations up to 10 mg/L, except for 48 hpf 0.5 mg/L. The increase in HBPM was the greatest between 0 and 10 mg/L (24 hpf: 16.75%, 48 hpf: 8.23%, 72 hpf: 8.09%). HBPM decreased most at 6000 mg/L (mean ± SD, 24 hpf: 55.55 ± 12.03, 48 hpf: 119.73 ± 27.82, 72 hpf: 146 ± 157.15 HBPM) relative to controls (*p* < 0.01; Supplementary Table S6).

### Embryo body burden

Exposure media at 0–20,000 mg/L HFPO-DA had a mean percent deviation of 8.34% ± 6.21 (Supplementary Table S7). Based on three pooled replicates of X embryos per replicate (1.026 g dry weight) exposed to 4000 mg/L HFPO-DA, the body burden was 479 ± 43 mg/kg HFPO-DA (Table 1); the bioconcentration factor was 0.12 L/kg dry weight. No HFPO-DA was detected in control exposure media or control embryo tissues.

**Table 1:** HFPO-DA concentration measurements in the zebrafish embryo tissue and media

HFPO-DA concentration in zebrafish embryo (mg/kg)					
Body burden	Mass (g)	Rep 1	Rep 2	Rep 3	Mean ± SD
0 mg/L exposure	0.702	ND	ND	ND	ND
4000 mg/L exposure	1.026	442	527	469	479 ± 43
HFPO-DA concentration in media (mg/L)					
Concentration in media	0 h	24 h	48 h	72 h	Mean ± SD
4000 mg/L exposure	4080	4420	3730	3880	4028 ± 298.37
Bioconcentration factor (BCF, L/kg dry weight): 0.12					

Tissue was collected from pooled zebrafish embryos exposed to 0 mg/L or 4000 mg/L HFPO-DA from 3 to 72 hpf, homogenized, and subsampled for replicates. Exposure media for analysis closely followed media treatment throughout embryo exposure, with the exclusion of embryos. Media for analysis was created, allowed to age for 72 h at 28.5 °C, and had 50% of media exchanged at 24 h and 48 h. Method detection limit (MDL) is 1 ng/g (fish) and 10 ng/L (media/water). ND represents no data

### *Shallow RNA-Seq* analysis of gene expression

**Fig. 4:**
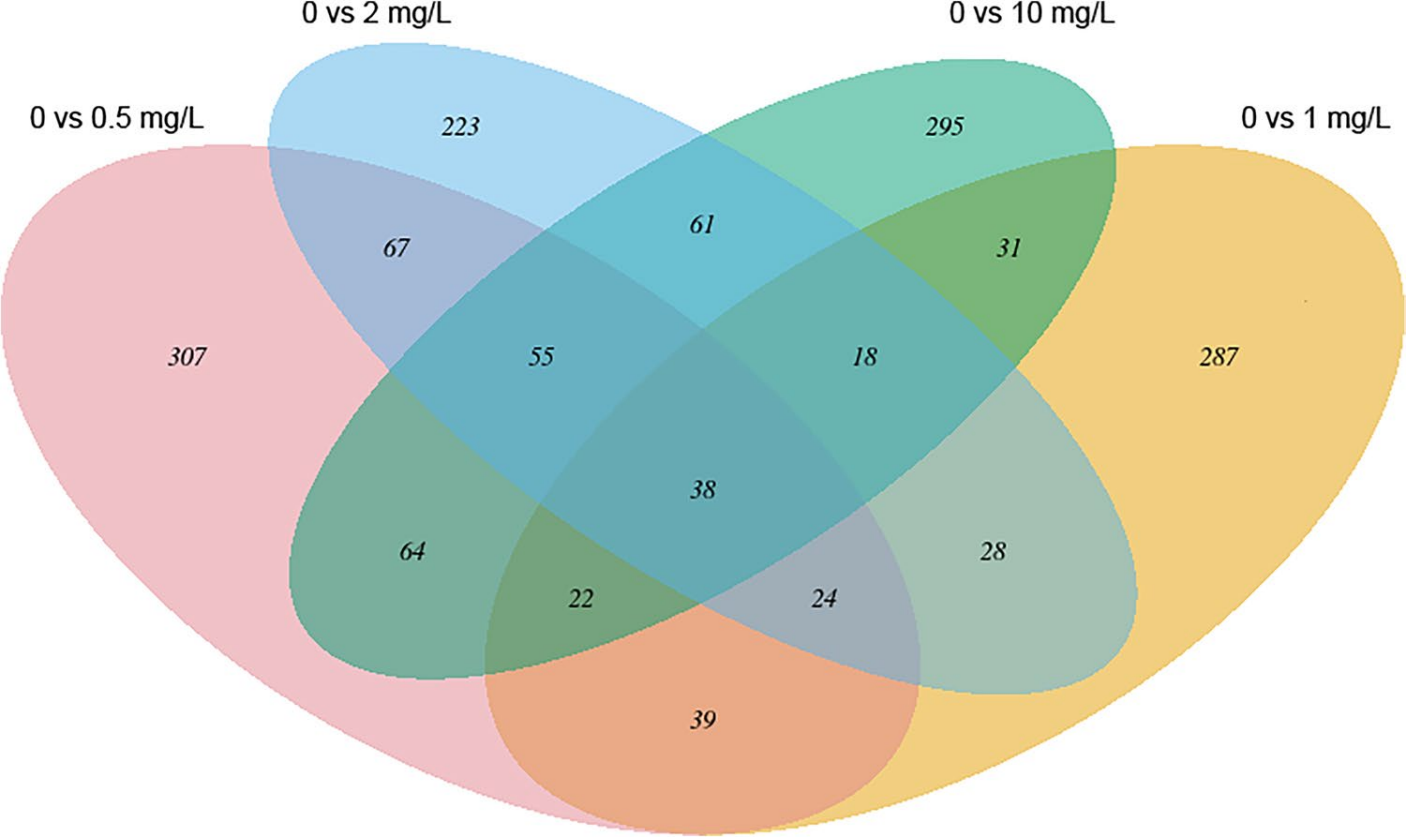
Thirty-eight genes were differentially expressed across all exposures relative to control at 72 hpf. Of 9465 transcripts analyzed, 1559 (16.5%) were differentially expressed in at least one exposure relative to the control. Most of the DEGs were found in only one exposure

Of 9465 genes analyzed, 16.47% (1559 genes) were differentially expressed in embryos exposed to 0.5 mg/L, 1 mg/L, 2 mg/L, or 10 mg/L HFPO-DA (*q* > 0.9, FDR < 0.1; Fig. 4, Supplementary Table S1); relative to control embryo gene expression, 41% of differentially expresses genes (DEGs) were downregulated and 59% were upregulated. Most DEGs (616 genes) were in the lowest HFPO-DA exposure of 0.5 mg/L, while the 1 mg/L exposure had the least (487 genes). The percentage of DEGs per individual exposure and percentage overlaps are presented in Fig. 4, and the ten highest and ten lowest expressed genes in each exposure are presented in Supplementary Fig. S2 and Table S2.

**Fig. 5:**
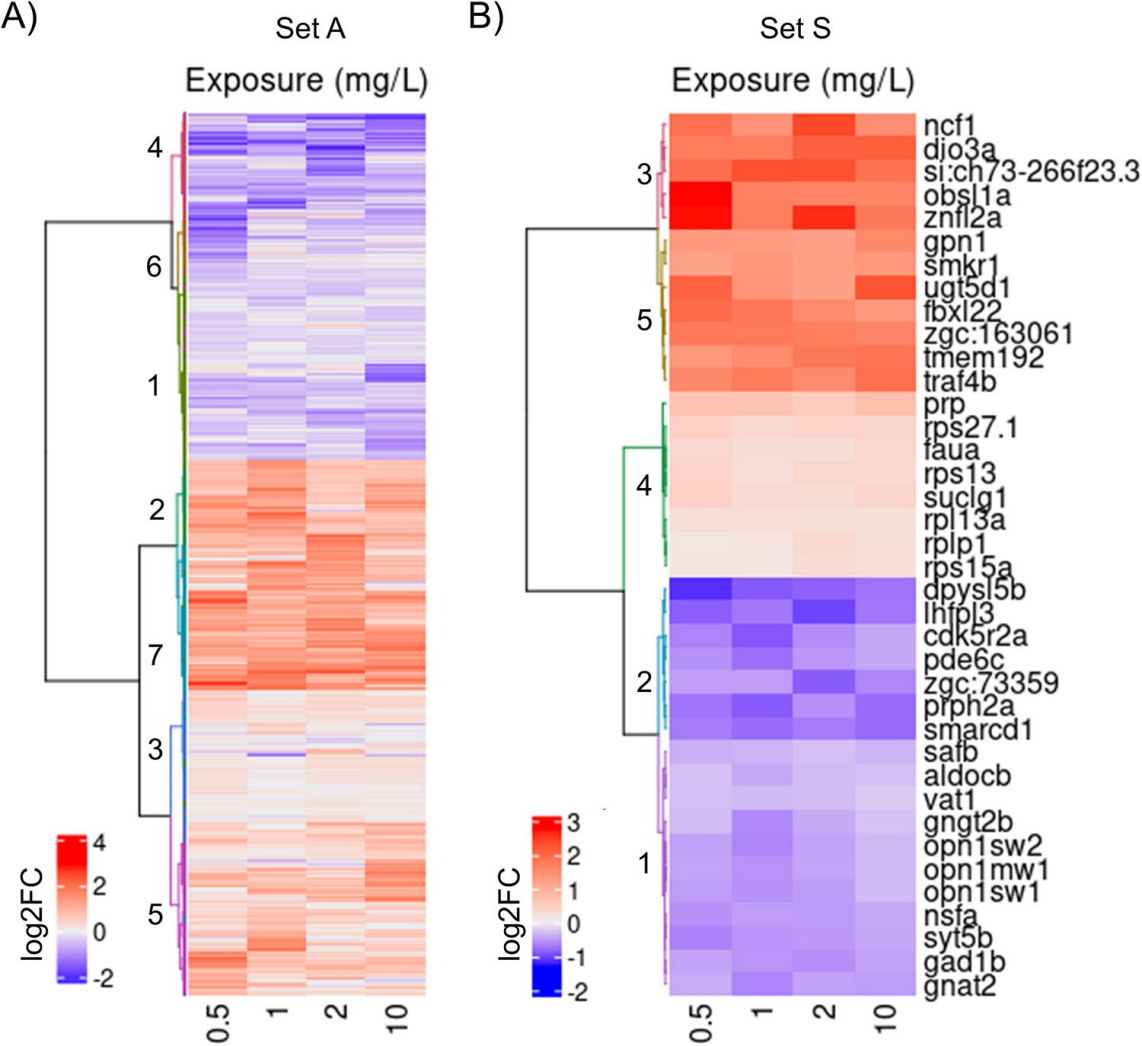
Clustering of the differentially expressed gene (DEG) at 0.5 mg/L, 1 mg/L, 2 mg/L, and 10 mg/L HFPO-DA exposures in zebrafish embryos at 72 HPF. (**A**) 1559 DEGs were identified in at least one exposure relative to control; (**B**) 38 DEGs (listed on the right) were found in all exposures relative to control (FDR < 0.1). Each column represents an exposure, and each row represents a gene. Colors reflect log_2_ fold changes, calculated using 3–4 biological replicates in the NOISeq R package. Red indicates high expression levels, and blue indicates low. Agglomerative hierarchical clustering of genes was performed using Ward’s method

DEGs in at least one exposure (1559 genes) are designated as set A (Fig. 5A), while DEGs shared across all four exposures are designated set S (Fig. 5B). Hierarchical clustering outlines seven clusters within set A and five clusters in set S (Fig. 5, Supplementary Tables S3, S4, S5, and S6). Cluster A1 (349 genes, 94.3% downregulated) is enriched for genes related to perception: A subset of 11 downregulated genes forms cluster S1, which is enriched for the detection and response of abiotic, chemical, and light stimuli; another subset of 7 downregulated genes form cluster S2, which is enriched for the development of eye and nervous systems. Upregulated clusters A3 (237 genes, 85% upregulated) and S4 (8 genes, 100% upregulated) are enriched for gene expression regulation and macromolecular biosynthesis. Cluster A2 (275 genes, 98.5% upregulated) and cluster S3 (5 genes, 100% upregulated) have DEGs implicated in defense responses to fungus and respiratory burst. Cluster S5 DEGs (6 genes, 100% upregulated) regulate actin filament-based movement, cardiac muscle cell contraction, and activation of NF-kappaB-inducing kinase activity. Clusters A4 (172 genes, 95.6% upregulated), A6 (93 genes, 100% downregulated), and A7 (129 genes, 100% upregulated) were not enriched for any terms at an FDR adjusted *p* < 0.05*.*

**Fig. 6:**
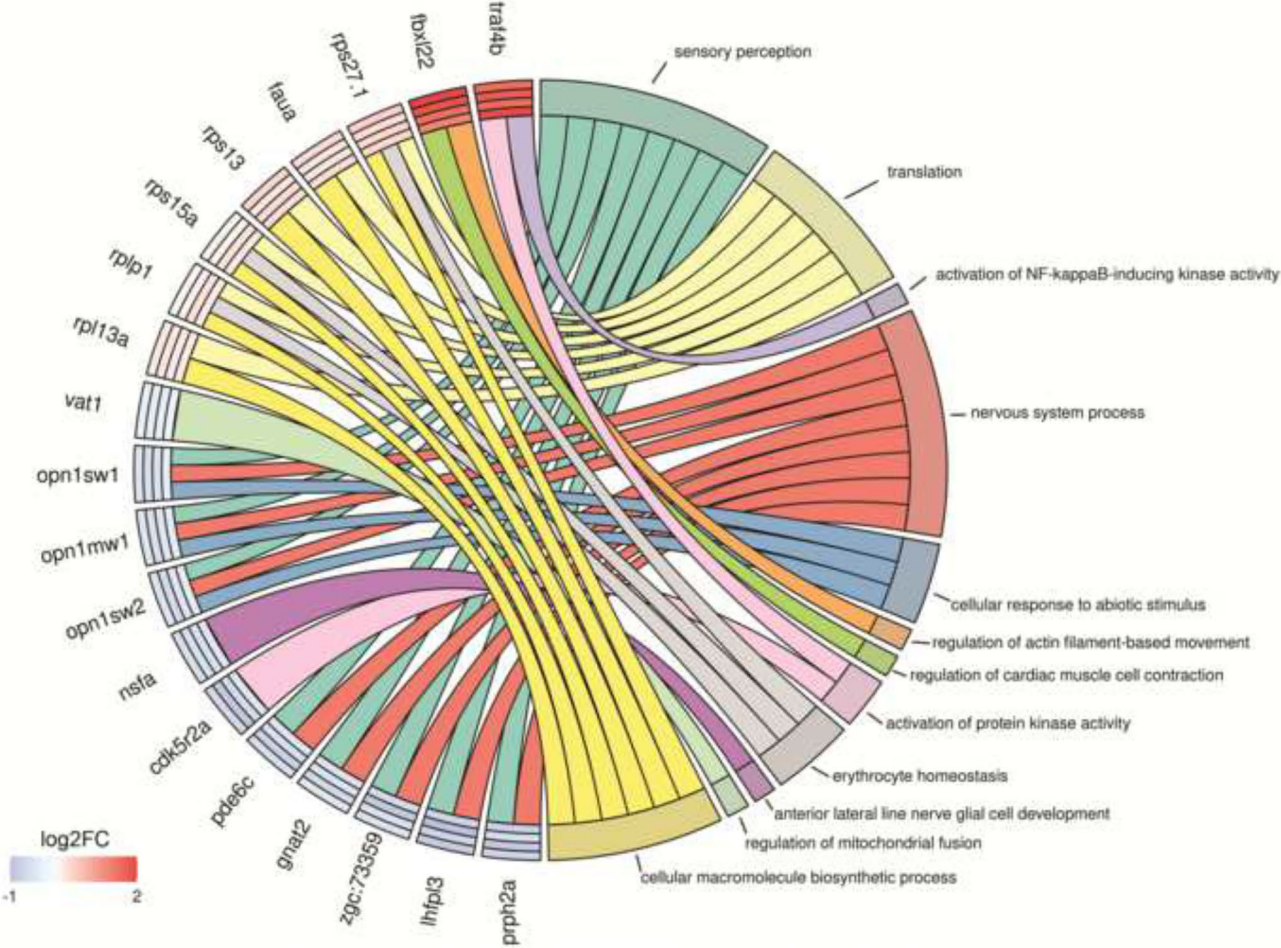
Chord diagram showing relationships between gene ontology (GO) terms and DEGs. Enrichment analysis was performed on the set of DEGs found in all zebrafish embryo exposure (0.5 mg/L, 1 mg/L, 2 mg/L, and 10 mg/L) relative to the control. Select GO terms in all exposures (FDR corrected *p* < 0.05) are shown. Genes are listed with log_2_ fold changes (0.5 mg/L, 1 mg/L, 2 mg/L, and 10 mg/L listed from outer to inner circle, legend colors only specify fold change)

Gene ontology analysis revealed enriched biological processes in set S. Three genes, *aldocb* (downregulated), *suclg1* (upregulated), and *ugt5d1* (upregulated), are involved in metabolic processes; *aldocb* and *suclg1* encode key proteins in cellular respiration, while *ugt5d1* is involved in drug metabolism. Six downregulated genes (*dpysl5b*, *gad1b*, *vat1*, *nsfa*, *lhfpl3*, *smarcd1*) are involved in neurotransmission or neurogenesis, and ten downregulated genes (*pde6hb*, *pde6c*, *prph2*, *gnat2*, *gngt2b*, *opn1mw1*, *opn1sw1*, *opn1sw2*, *syt5b*, *cdk5r2a*, *obsl1a*, *dio3a*) are involved in eye development or the phosphodiesterase 6 (PDE6) phototransduction cascade. Seven upregulated genes (*znfl2a*, *fbxl22*, *col22a1*, *ncf1*, *rplp1*, *rps15a*, *rps27.1*) play a role in muscle contraction, regulation of vascular stability, or hemopoietic development. Major enrichment terms and associated DEGs are shown in Fig. 6.

Expression of 11 genes at 0 mg/L, 0.5 mg/L, and 10 mg/L HFPO-DA exposures were compared between shallow RNA sequencing and qRT-PCR (Supplementary Fig. S3): Nine genes’ expression patterns correlated, and two did not (*obsl1a, dio3a*; Supplementary Table S7). *R*^2^ values of TMM-normalized shallow RNA-Seq expression to qRT-PCR ∆Ct values are 0.68, 0.737, and 0.725 at 0 mg/L, 0.5 mg/L, and 10 mg/L HFPO-DA exposure, respectively.

## Discussion

This study assessed the toxicity potential of the emerging PFAS HFPO-DA during animal development. High concentrations of HFPO-DA exposures resulted in morphological changes, including scoliosis and edema at lethal HFPO-DA concentrations, increases in HBPM, and significant gene expression changes in visual and cardiovascular systems at sublethal concentrations among zebrafish embryos assayed from 3 to 72 hpf. Data were derived from pH-neutralized HFPO-DA exposures to avoid confounding effects of acid toxicity; the pH of exposure media was not discussed in prior studies, which may explain variations in reported PFAS lethality and adverse effects (Gebreab et al. 2020; Wasel et al. 2020). We utilized the cost-effective shallow RNA sequencing method (Atallah et al. 2013; Sholder et al. 2020; C. Ye et al. 2018) to analyze the expression of 9465 genes at four exposure concentrations (0.5 mg/L, 1 mg/L, 2 mg/L, and 10 mg/L) of HFPO-DA. Our results reveal overlapping sets of enrichment terms consistent across exposures, including altered expression of genes expressed in the heart and vascular tissue, suggesting cardiovascular toxicity of HFPO-DA, like its predecessor PFOA (Salimi et al. 2019).

To evaluate HFPO-DA toxicity, we first determined the lethal concentrations in developing zebrafish embryos. The untitrated LC_50_ corresponds to an estimated pH of 4.3 (Supplementary Fig. S1 and Table S1). We derived a stronger correlation of embryo survival with acidity (*R*^2^ = 0.80) than with the HFPO-DA concentration (*R*^2^ = 0.65) in untitrated HFPO-DA media. Therefore, the LC50 in untitrated HFPO-DA of 51 mg/L, which agrees with the reported LC_50_ of 170 µM (56 mg/L) (Satbhai et al. 2022), is more likely due to the low pH rather than HFPO-DA-specific exposure, highlighting the pH effect of PFAS on zebrafish embryo mortality and the need to neutralize pH in aqueous exposure experiments (Wasel et al. 2020).

LC_50_ of pH-neutralized HFPO-DA is 7651 mg/L (95% CI = 5976–8080 mg/L) in zebrafish embryos exposed from 3 to 72 hpf. We utilized much higher concentration exposures than measured in environmental samples and occupational exposure hazards (Olsen et al. 2007) to establish lethal levels of HFPO-DA in zebrafish embryos and to explore differential gene expression using shallow RNA-Seq. LC_50_s of PFAS were reported in the thousands of milligrams per liter, including pentafluorobenzoic acid (PFBA) at 13,795 mg/L (96 hpf) (Godfrey et al. 2017), and PFBA and perfluorobutanesulfonic acid (PFBS) at greater than 3000 mg/L (120 hpf) (Hagenaars et al. 2011). Such LC_50_s are much higher than those of phased-out long-chain PFAS, including PFOA (473 mg/L, 96 hpf) or PFOS (70.17 mg/L, 120 hpf; 2.2 mg/L, 120 hpf) in similar zebrafish embryo studies (Ding et al. 2012; Godfrey et al. 2017; Huang et al. 2010), suggesting lower HFPO-DA toxic potential to zebrafish embryos than its long-chain predecessors. Furthermore, we analyzed the body burdens of embryos exposed to 4000 mg/L HFPO-DA until 72 hpf and determined a bioconcentration factor of 0.12, similar to reported low BCF values of 0.29 to 0.49 after 144 hpf zebrafish embryo exposures to 25.1–44.8 µM HFPO-DA (Gaballah et al. 2020). The low BCF value indicates that HFPO-DA does not bioaccumulate under our experimental conditions.

The incidence of malformation in zebrafish embryos was evident at nonlethal (1000 mg/L) to 100% lethal (20,000 mg/L) HFPO-DA exposures (Fig. 2). Observed spinal deformities, edema, and hemorrhaging are common phenotypes in zebrafish developmental toxicity (Lee et al. 2020; Martínez et al. 2019) and are often reported in zebrafish PFAS exposures (Gebreab et al. 2020; Huang et al. 2010; Martínez et al. 2019). Spinal deformations were the dominant malformation observed in zebrafish embryos exposed to 1000–20,000 mg/L HFPO-DA. Kyphosis at the head-trunk junction not qualified as spinal curvature may be due to developmental delay, as the development between the pectoral fin (60 hpf) and protruding mouth (72 hpf) involves a gradual straightening of the head-trunk angle (from 55° to 25°), extension of the pectoral fin, protrusion of the jaw, and decrease in yolk-size (Kimmel et al. 1995). Malformations were evident in all exposures above 1000 mg/L; only 12.8% of hatched embryos at 1000 mg/L exhibited malformation compared to 83.3% at 4000 mg/L. Malformations were significant in 4000–20,000 mg/L exposures (Fig. 2), and their incidence increased with higher exposure concentrations (*R*^2^ = 0.6375), indicating a positive dose–response relationship.

Heart rate is a common co-indicator of cardiac disruption when reported with morphological effects (Craig et al. 2006; Kuhota et al. 1997). Zebrafish are an established model for human cardiac disease (den Hoed et al. 2013; Gierten et al. 2020; Milan et al. 2003), and their cardiac rhythm and regulation via electrical conduction are similar to those in humans (Arnaout et al. 2007; Gauvrit et al. 2022; Nemtsas et al. 2010). Interestingly, HBPM increased in a dose-dependent manner at 0.5–10 mg/L exposures, and HBPM decreased in a dose-dependent manner at 1000–6000 mg/L (Fig. 3). Tachycardia at low-level chemical exposures followed by bradycardia at higher levels has previously been observed (Han et al. 2021; Maeda et al. 2021) and may indicate a hormetic cardiac response to HFPO-DA exposure (Sampurna et al. 2019). Tachycardia and bradycardia can affect the regulation of cardiac muscular contractility (Søndergaard et al. 2015) or circulatory demand in response to respiratory and metabolic stress (Miller et al. 2014). Co-occurrence of pericardial edema and hemorrhaging with bradycardia at these concentrations and increased mortality from 6000 to 20,000 mg/L strongly indicate cardiovascular defects (Duan et al. 2013; Liu et al. 2017).

During normal embryo development, the transcriptome landscape is dynamic (H. Yang et al. 2013), with significant changes in the gene expression marking distinct stages of embryo development. To better understand the mechanisms of HFPO-DA-induced morphological and physiological effects, we utilized shallow RNA-Seq analysis. To compensate for expected false negatives due to low sequencing depth, we used NOISeq, a nonparametric tool with high sensitivity at low sequencing depth (C. R. Williams et al. 2016), and applied a threshold *q* > 0.9 (equivalent to FDR < 0.1). Seventy-one percent of detected DEGs were significant in only one exposure relative to control (Fig. 4). Surprisingly, the highest number of DEGs was detected at the lowest exposure concentration, possibly indicating changing tolerance (Andrade et al. 2017) to HFPO-DA across the exposures, or a non-monotonic dose response (Z. H. Li et al. 2013). The ten most upregulated and downregulated genes in each exposure share functional similarities: Most of these genes are related to eye, neural, and vascular systems, metabolism/biosynthesis, gene expression regulation, and development (70%, 70%, 75%, and 80% in 0.5 mg/L, 1 mg/L, 2 mg/L, and 10 mg/L, respectively). Set A (1559 DEGs) and set S (38 DEGs) share GO terms (74 terms; 29.4% of set A, 55.22% of set S; Supplementary Tables S3, S4, S5, and S6) despite set S constituting only 2.4% of the total DEGs (Fig. 5). Set S was further analyzed since it consists of DEGs across all exposures.

Visual sensation begins with the excitation of opsins (such as *opn1mw1*, *opn1sw1*, and *opn1sw2*), which activate photoreceptor-specific G-protein transducin subunits, encoded by *gnat2* and *gngt2b* (Lagman et al. 2015; Tsujikawa and Malicki 2004). This initiation of the signaling cascade is followed by the activation of the PDE6 complex by *gnat2.* Zebrafish embryos are capable of shadow-induced startle response at 72 hpf once opsin expression has spread to cones (Easter and Nicola 1996). Furthermore, light-response hypoactivity has been reported in zebrafish exposed to 0.76 µM HFPO-DA (250 µg/L) until 120 hpf (Rericha et al. 2021). Downregulation of *dio3a* is notable, as knockdown of *dio3a* previously demonstrated delays in eye development through the regulation of thyroid hormone (Bagci et al. 2015; Heijlen et al. 2014; Houbrechts et al. 2016). Downregulation of opsin expression and PDE6 complex subunits during light sensitivity development suggests a mechanism by which HFPO-DA could disrupt visual sensory response, while the downregulation of *dio3a* suggests the developmental delay is responsible for the decreased expression of genes related to the visual system. Light-response assays during advanced developmental stages are needed to confirm these genotype–phenotype correlations induced by HFPO-DA exposures.

Glutamate decarboxylase *gad1b*, which is downregulated in exposed embryos, acts downstream of the Krebs (tricarboxylic acid) cycle to produce gamma-aminobutyric acid (GABA) (Buddhala et al. 2009). *gad1b* is regularly differentially expressed when GABAergic activity is affected by chemical exposure (Filippi et al. 2014; Yu et al. 2017), and its knockdown is reported to induce craniofacial defects in zebrafish embryos (O’Connor et al. 2019). Downregulated genes *dpysl5b* and *nsfa* are essential for axon development (Takeuchi et al. 2017) and the neuroendocrine system (Kurrasch et al. 2009; Woods et al. 2006), respectively. Decreased *gad1b*, *nsfa*, and *dpys5b* expression may indicate HFPO-DA interference with embryo neurotransmission at 72 hpf via GABA biosynthesis and neural development inhibition. Although exposures up to 26.4 mg/L HFPO-DA until 144 hpf suggested no neurodevelopmental toxicity (Gaballah et al. 2020), hypoactivity in dechorionated zebrafish embryos exposed to 250 µg/L HFPO-DA until 120 hpf suggests neurodevelopmental effect (Rericha et al. 2021).

Upregulated gene *fbxl22* encodes the substrate binding subunit of SCF-E3 ligase (Hughes et al. 2020) responsible for the ubiquitination and regulation of sarcomeric proteins. *fbxl22* overexpression induces atrophy and degradation of essential sarcomeric proteins in mouse skeletal muscle (Hughes et al. 2020), while knockdown in zebrafish embryos severely impairs muscle contraction (Spaich et al. 2012). Furthermore, *fbxl22* overexpression and subsequent sarcomere disassembly have been described in cardiomyocyte regeneration at sites of injury (Beisaw et al. 2020). *col22a1* and *znfl2a* are associated with the development and maintenance of zebrafish vascular systems (Qian et al. 2005); overexpression of *col22a1* has been shown to rescue an increased vascular permeability phenotype in homozygous *col22a1* mutants (Ton et al. 2018)*. col22a1* knockdown has further been shown to induce muscular dystrophy and muscle fiber detachment in zebrafish embryos (Charvet et al. 2013). Although the DEGs in this study provide no direct evidence for HBPM increase, the upregulation of *fbxl22* may indicate cardiac stress and vascular effect via upregulation of *col22a1 and znfl2a*, while *col22a1* upregulation may be relevant to scoliosis phenotype at 4000 to 12,000 mg/L HFPO-DA exposures. Increased HBPM at 2 mg/L and 10 mg/L HFPO-DA may be a manifestation of cardiovascular stress, but the mechanism of action is unclear without additional loss-of-function/gain-of-function experiments coupled with histological and angiographic data. Clusters of downregulated vision and neurogenesis genes and upregulated cardiovascular genes (Fig. 5) suggest system-specific adverse effects of HFPO-DA exposure.

High-level exposures toxicity studies of novel chemicals or unknown mechanisms of action are necessary to determine toxicological potential, derive lethal concentration parameters, identify morphological, physiological, and molecular toxicity targets, and anticipate adverse effects during potential acute and cumulative chronic occupational and environmental exposures (Kakade et al. 2020). Both molecular mechanisms and the relationships between exposure concentration and toxic effects with time need to be considered to understand the toxicity of chemicals to a developing organism (Tennekes and Sánchez-Bayo 2013). Novel yet ubiquitous chemicals such as HFPO-DA potentially having non-specific receptor binding or involving slowly reversible binding to some receptors that do not contribute to toxicity may be time-dependent; however, their effects may also depend primarily on the exposure concentration, with time playing a minor role. Consequently, the mechanism of toxicity has important implications for risk assessment (Tennekes and Sánchez-Bayo 2013). Conventional toxicity testing relies on extensive observations of phenotypic endpoints in vivo. The utility of novel materials and chemicals mandates a better understanding of the morphological, physiological, genetic, and molecular targets and changes occurring in exposed biological systems.

Our experimental design has several limitations: Pooling whole embryos and larvae does not address individual variation or tissue- or cell type-specific effects, likely resulting in a loss of tissue-specific markers and organ-targeting toxicity of HFPO-DA. Nonetheless, several genes affecting visual and cardiovascular systems suggest HFPO-DA tissue-specific effects might be of further interest. While generally, there is a significant correlation between gene transcription and protein synthesis (Maier et al. 2009), we did not assess the HFPO-DA effects post-transcriptionally. HBPM was the only in vivo quantified physiological phenotype combined with gene expression at 0.5 mg/L, 1 mg/L, 2 mg/L, and 10 mg/L HFPO-DA. Because increased lethality, malformation, and neurodevelopmental toxicity with extended exposures to other PFAS (past 80 hpf or 96 hpf) have been reported (Gebreab et al. 2020; Huang et al. 2010; Mylroie et al. 2021), we recommend that future studies focus on sublethal concentration and extend the exposure time to swimming and feeding young larvae, thus allowing for behavioral assays. Histology or in situ hybridization analyses are complements to gene expression when evaluating HFPO-DA-induced organ-specific genotype–phenotype correlations.

Transcriptomics allows for detecting organisms’ responses to environmental, chemical, and physical agents by directly measuring the molecular alterations (Kinaret et al. 2020; Serra et al. 2020). In this study, we intergraded classical in vivo developmental toxicology with toxicogenomics to the characterization of the mechanism of action (MOA) and molecular targets of HFPO-DA. We report altered HBPM, morphological changes including scoliosis and edema at lethal HFPO-DA concentrations, and effects on the expression of genes relevant to the development of nervous and cardiovascular systems in zebrafish embryos assayed from 3 to 72 hpf. While we report HFPO-DA-induced alterations at extremely high exposures that are 10^4^- to 10^9^-fold higher than levels detected at sites contaminated with HFPO-DA, our study identifies adverse effects, corresponding molecular targets (Alexander-Dann et al. 2018), and biological pathways affected by HFPO-DA during early zebrafish development. Adverse effects and altered phenotypes in this study are observed only at extremely high acute exposure concentrations and were not detected during low-level exposures (Gaballah et al. 2020). Our data on exposure time, dose, complex endpoint selection, and potential targets of toxicity during animal development are important to better understand and predict HFPO-DA chemical toxicity potential. To our knowledge, this is the first study to examine the effects of HFPO-DA exposure on heart physiology and gene expression through shallow RNA-Seq. As the next phase of further assessing HFPO-DA toxicity potential, we recommend longer chronic exposures at environmentally relevant concentrations and considering phenotypes and molecular targets identified in our study.

## Supplementary Information

Below is the link to the electronic supplementary material.

Supplementary file1 Supplementary Tables, shallow RNA-Seq data tables (XLSX 416 KB)

Supplementary file2 Supplementary Materials, including data tables and figures (DOCX 1803 KB)

## Data Availability

The shallow RNA-Seq datasets generated and analyzed during the current study are available in NCBI’s Gene Expression Omnibus and are accessible through GEO Series accession number GSE198976 (https://www.ncbi.nlm.nih.gov/geo/query/acc.cgi?acc=GSE198976). All datasets used and analyzed during the current study are available from the corresponding author upon reasonable request.

## Abbreviations

*HBPM*: Heart beats per minute
*hpf*: Hours post-fertilization
*HFPO-DA*: Hexafluoropropylene oxide-dimer acid
*PFAS*: Per- and polyfluoroalkyl substances
