# Supplementary material for: Toxicity assessment of hexafluoropropylene oxide-dimer acid on morphology, heart physiology, and gene expression during zebrafish (Danio rerio) development
Source: Environ Sci Pollut Res Int. 2022 Dec 3;30(12):32320–36. doi: 10.1007/s11356-022-24542-z (PMC10017623; doi:10.1007/s11356-022-24542-z)
Supplement: Supplementary file 2 — Supplementary file2 Supplementary Materials, including data tables and figures (DOCX 1803 KB) [file 11356_2022_24542_MOESM2_ESM.docx]

**Supplementary Table 1, Chemical structures of select poly- and per-fluoroalkyl substances.**

| **Chemical** | **Structure** |
| --- | --- |
| PFOA | 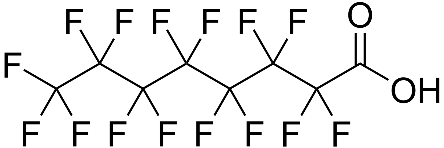 |
| HFPO-DA | 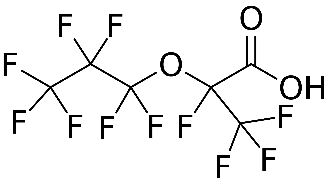 |
| PFNxS | 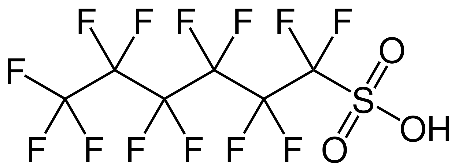 |
| PFNA | 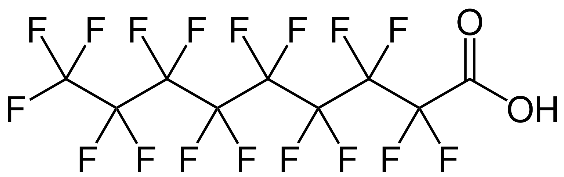 |
| PFBS | 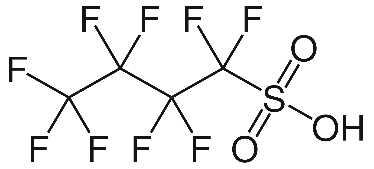 |
| PFBA | 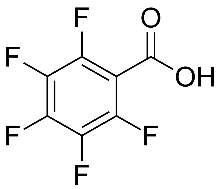 |

**Supplementary Table 2, Titrated survival.**

| **Exposure (mg/L)​** | **Total Embryos​** | **% Mean Survival ± SD​** | **% Unhatched​** | **Mean Morphology Score​ ± SD** |
| --- | --- | --- | --- | --- |
| 0​ | 120​ | 92.86 ± 7.26​ | 4.17​ | 1 ± 0​ |
| 1000​ | 60​ | 86.67 ± 10.33​ | 1.67​ | 1.16 ± 0.51 |
| 4000​ | 60​ | 68.33 ± 21.37​ | 11.67​ | 2.37 ± 0.97​ |
| 6000​ | 60​ | 63.33 ± 13.66​ | 15​ | 2.64 ± 1.22​ |
| 8000​ | 130​ | 43.85 ± 27.55​ | 14.62​ | 3.04 ± 0.93​ |
| 10000​ | 80​ | 36.25 ± 29.73​ | 11.25​ | 3.21 ± 0.77​ |
| 12000​ | 80​ | 8.75 ± 8.35​ | 2.5​ | 3.17 ± 0.75​ |
| 16000​ | 80​ | 10 ± 16.035​ | 3.75​ | 3.75 ± 0.4​6 |
| 20000​ | 90​ | 1.11 ± 3.33​ | 1.11​ | 3* |

*Only one embryo survived in the 20000 mg/L exposure.

Number of embryos, percent mean survival, mean morphology scores, and percent unhatched zebrafish embryos from titrated HFPO-DA exposures are shown. Morphology scores were calculated from surviving larvae. All exposure media were titrated to pH 6.6 – 7.6. Zebrafish embryos were exposed from 3 to 72 hpf; survival and morphology were recorded at 72 hpf.

**Supplementary Table 3, qRT-PCR primers.**

| **Gene​** | **Accession​** | **Forward (F) /Reverse (R)​** | **Primers (5' - 3')​** |
| --- | --- | --- | --- |
| *cdk5r2a​* | [NM_001017569.1](https://www.ncbi.nlm.nih.gov/nucleotide/NM_001017569.1?report=genbank&log$=nuclalign&blast_rank=27&RID=VZ235ENK013)​ | (F)​ | ACCTGGACCGCTAAGTTTGG​ |
|  |  | (R)​ | AACGCTCCTCACACCTTCAG​ |
| *dio3a​* | [NM_001256003.1](https://www.ncbi.nlm.nih.gov/nuccore/NM_001256003)​ | (F)​ | TAAAAGGGAAGCGGAGCGTC​ |
|  |  | (R)​ | CGGGAGAATGAGCAGACACA​ |
| *dpysl5b​* | [NM_001020515.1](https://www.ncbi.nlm.nih.gov/nucleotide/NM_001020515.1?report=genbank&log$=nuclalign&blast_rank=2&RID=VZ0J9B7W013)​ | (F)​ | CTGGATCTGGGCATCAGTGG​ |
|  |  | (R)​ | GCCTGGACCACCTTACCTTG​ |
| *fbxl22​* | [NM_001013279.2](https://www.ncbi.nlm.nih.gov/nucleotide/NM_001013279.2?report=genbank&log$=nuclalign&blast_rank=1&RID=VZ2JS0TM013)​ | (F)​ | GAGTGGACTTTTGCCGCAATGTG​ |
|  |  | (R)​ | AGGTCACAACGGTCAGTCAAGAG​ |
| *gpn1​* | [NM_001003633.1](https://www.ncbi.nlm.nih.gov/nucleotide/NM_001003633.1?report=genbank&log$=nuclalign&blast_rank=4&RID=VZ31KUWZ01R)​ | (F)​ | GACACTCCAGGGCAAATAGAGGTG​ |
|  |  | (R)​ | CAGGGAAAAGAAGAGGCCAGTGC​ |
| *lhfpl3​* | [NM_001003444.1](https://www.ncbi.nlm.nih.gov/nucleotide/NM_001003444.1?report=genbank&log$=nuclalign&blast_rank=7&RID=VZ589FTA013)​ | (F)​ | CAGAGGCCGCCAAGATCTAC​ |
|  |  | (R)​ | CCGATCCAGTAGGGCTGAAC​ |
| *ncf1​* | [NM_001030071.2](https://www.ncbi.nlm.nih.gov/nucleotide/NM_001030071.2?report=genbank&log$=nuclalign&blast_rank=3&RID=VZ5HE6Y4013)​ | (F)​ | CACCACCACCAAGAAGGTCG​ |
|  |  | (R)​ | GCTCTGTTTCCGGTAGCTGT​ |
| *prph2a​* | [NM_131566.1](https://www.ncbi.nlm.nih.gov/nucleotide/NM_131566.1?report=genbank&log$=nuclalign&blast_rank=10&RID=VZ5VS3PV013)​ | (F)​ | ATGGAGTTCCGATGCTGTGG​ |
|  |  | (R)​ | GCTGCTGATTCGGTCTTTGAC​ |
| *smarcd1​* | [NM_198358](https://www.ncbi.nlm.nih.gov/nuccore/NM_198358)​ | (F)​ | CATGCCAGTAATGGACCCGT​ |
|  |  | (R)​ | TTCTTGGCATGTTGGTTGCG​ |
| *traf4b​* | [NM_212817](https://www.ncbi.nlm.nih.gov/nuccore/NM_212817)​ | (F)​ | AGGAGTGTTCACATGCCCAGAAG​ |
|  |  | (R)​ | AGCGGATGGGCAAAGAGAGAATC​ |
| *ube2a​* | [NM_201273](https://www.ncbi.nlm.nih.gov/nuccore/NM_201273)​ | (F)​ | GCTGTTGTGTTGTGGTCTCC​ |
|  |  | (R)​ | CGAGCAGCCAACATGTGAAT​ |
| *tmem50a​* | [NM_213529.2](https://www.ncbi.nlm.nih.gov/nucleotide/NM_213529.2?report=genbank&log$=nuclalign&blast_rank=4&RID=VZ8MA3DM016)​ | (F)​ | GTAGGAGACGGAAGACTGCG​ |
|  |  | (R)​ | TTCTGCTGATCTGACCTTGAG​ |
| *ef1a​* | [NM_131263](http://ahttps/zfin.org/ZDB-GENE-990415-52#marker-relationship)​ | (F)​ | CTACCCTCCTCTTGGTCGCT​ |
|  |  | (R)​ | GGAACGGTGTGATTGAGGGAA​ |
| *actb1​* | [NM_131031](https://www.ncbi.nlm.nih.gov/nuccore/NM_131031)​ | (F)​ | TTCAGTGCACGCTGAGAAGAT​ |
|  |  | (R)​ | CAACCATCACTCCCTGATGTC​ |
| *ugt5d1​* | [NM_001177496.1](https://www.ncbi.nlm.nih.gov/nuccore/NM_001177496)​ | (F)​ | AGCAGGGGGAGTGTTTTTAGCAC​ |
|  |  | (R)​ | TCGGAGATGGTGCGATAGCAAAG​ |

**Supplementary Table 4, Untitrated survival.**

| **Exposure (mg/L)** | **Trials​** | **Total Embryos​** | **Mean % Survival ± SD​** | **Mean pH ± SD​** |
| --- | --- | --- | --- | --- |
| 0​ | 17​ | 390​ | 94.38 ± 4.88​ | 6.79 ± 0.13​ |
| 10​ | 8​ | 180​ | 95.94 ± 6.81​ | 5. 70 ± 0.79​ |
| 25​ | 12​ | 275​ | 85.83 ± 14.02​ | 5.08 ± 1.01​ |
| 50​ | 8​ | 190​ | 59.79 ± 31.34​ | 4.30 ± 0.74​ |
| 60​ | 3​ | 60​ | 35 ± 48.22​ | 4.32 ± 1.20​ |
| 75​ | 9​ | 247​ | 24.28 ± 40.29​ | 3.97 ± 0.75​ |
| 100​ | 15​ | 354​ | 22.48 ± 37.05​ | 3.69 ± 0.31​ |
| 150​ | 11​ | 264​ | 12.73 ± 28.67​ | 3.43 ± 0.21​ |

Numbers of trials, percent survival, and pH of zebrafish embryos exposures to untitrated HFPO-DA are shown. A minimum of 10 and a maximum of 75 embryos were exposed from 3 to 72 hpf. Survival and pH were recorded at 72 hpf.

**Supplementary Table 5, HBPM at low exposure concentrations.**

| **hpf​** | **Exposure (mg/L)​** | **Total Embryos​** | **Mean HBPM ± SD​** |
| --- | --- | --- | --- |
| 24​ | 0​ | 54​ | 63.89 ± 13.72​ |
| 24​ | 0.5​ | 53​ | 65.32 ± 12.5​ |
| 24​ | 1​ | 54​ | 67.89 ± 14.37​ |
| 24​ | 2​ | 56​ | 71.57 ± 13.72​ |
| 24​ | 10​ | 44​ | 74.59 ± 12.04​ |
| 48​ | 0​ | 52​ | 128.96 ± 11.48​ |
| 48​ | 0.5​ | 50​ | 128.36 ± 9.94​ |
| 48​ | 1​ | 54​ | 134.52 ± 12.14​ |
| 48​ | 2​ | 54​ | 138.56 ± 11.38​ |
| 48​ | 10​ | 43​ | 139.58 ± 12.53​ |
| 72​ | 0​ | 49​ | 153.71 ± 10.34​ |
| 72​ | 0.5​ | 50​ | 156.56 ± 13.01​ |
| 72​ | 1​ | 50​ | 160.12 ± 10.15​ |
| 72​ | 2​ | 49​ | 161.8 ± 13.2​ |
| 72​ | 10​ | 38​ | 166.16 ± 11.33​ |

HBPM were measured for zebrafish embryos exposed to 0.5 – 10 mg/L HFPO-DA in titrated exposure media at 24, 48, and 72 hpf. Zebrafish embryos were exposed from 3 to 72 hpf. Exposure to titrated HFPO-DA increased HBPM of embryos at all observed timepoints.

**Supplementary Table 6, HBPM at high exposure concentrations.**

| **hpf​** | **Exposure (mg/L)​** | **Total Embryos​** | **Mean HBPM ± SD​** |
| --- | --- | --- | --- |
| 24​ | 0​ | 29​ | 70 ± 8.59​ |
| 24​ | 1000​ | 28​ | 62.64 ± 8.43​ |
| 24​ | 4000​ | 24​ | 56.17 ± 8.59​ |
| 24​ | 6000​ | 22​ | 55.55 ± 12.03​ |
| 24​ | 8000​ | 13​ | 56.62 ± 7.18​ |
| 24​ | 10000​ | 9​ | 56.67 ± 6​ |
| 24​ | 12000​ | 1​ | 56 ± NA*​ |
| 48​ | 0​ | 29​ | 140.14 ± 12.11​ |
| 48​ | 1000​ | 29​ | 136.21 ± 12.52​ |
| 48​ | 4000​ | 27​ | 130.30 ± 18.96​ |
| 48​ | 6000​ | 22​ | 119.73 ± 27.82​ |
| 48​ | 8000​ | 13​ | 107.08 ± 13.51​ |
| 48​ | 10000​ | 9​ | 124 ± 9.38​ |
| 48​ | 12000​ | 1​ | 138 ± NA*​ |
| 72​ | 0​ | 29​ | 163.66 ± 167.97​ |
| 72​ | 1000​ | 29​ | 159.31 ± 163.55​ |
| 72​ | 4000​ | 27​ | 139.48 ± 150.54​ |
| 72​ | 6000​ | 22​ | 146 ± 157.15​ |
| 72​ | 8000​ | 13​ | 133.7 ± 140.67​ |
| 72​ | 10000​ | 9​ | 142.67 ± 148.47​ |
| 72​ | 12000​ | 1​ | 160 ± NA*​ |

HBPM were measured for zebrafish embryos exposed to 1000 – 12000 mg/L HFPO-DA in titrated exposure media at 24, 48, and 72 hpf. Zebrafish embryos were exposed from 3 to 72 hpf. HBPM were only recorded for surviving embryos; survival was significantly decreased from 4000 mg/L to 20000 mg/L. The lack of SD at 12000* mg/L and the lack of data at 20000 mg/L exposures were due to high mortality rates.

**Supplementary Table 7, Media HFPO-DA analysis**

| **Exposure Concentration**  **(mg/L)​** | **Time​-aged (h)** | **Measured Concentration (mg/L)​** | **Deviation​ (%)** |
| --- | --- | --- | --- |
| 0.5 | 0​ | 0.54​ | 8​ |
| 0.5 | 24​ | 0.47​ | 6​ |
| 0.5 | 48​ | 0.5​ | 0​ |
| 0.5 | 72​ | 0.44​ | 12​ |
| 1 | 0​ | 0.88​ | 12​ |
| 1 | 24​ | 1.3​ | 26​ |
| 1 | 48​ | 1.1​ | 10​ |
| 1 | 72​ | 0.95​ | 5​ |
| 2 | 0 ​ | 2.3​ | 16​ |
| 2​ | 24​ | 2.1​ | 4.5​ |
| 2​ | 48​ | 1.9​ | 5.5​ |
| 2 | 72​ | 2.2​ | 11.5​ |
| 10​ | 0​ | 9.1​ | 9​ |
| 10​ | 24​ | 9.9​ | 1​ |
| 10​ | 48​ | 11​ | 10​ |
| 10​ | 72​ | 8.5​ | 15​ |
| 1000​ | 0​ | 1070​ | 7​ |
| 1000​ | 24​ | 980​ | 2​ |
| 1000​ | 48​ | 920​ | 8​ |
| 1000​ | 72​ | 1100​ | 10​ |
| 4000​ | 0​ | 4080​ | 2​ |
| 4000​ | 24​ | 4420​ | 10.5​ |
| 4000​ | 48​ | 3730​ | 6.75​ |
| 4000​ | 72​ | 3880​ | 3​ |
| 6000​ | 0​ | 5670​ | 5.5​ |
| 6000​ | 24​ | 5400​ | 10​ |
| 6000​ | 48​ | 6030​ | 0.5​ |
| 6000​ | 72​ | 6340​ | 5.67​ |
| 8000​ | 0​ | 7460​ | 6.75​ |
| 8000​ | 24​ | 7520​ | 6​ |
| 8000​ | 48​ | 8090​ | 1.13​ |
| 8000​ | 72​ | 7810​ | 2.38​ |
| 10000​ | 0​ | 10300​ | 3​ |
| 10000​ | 24​ | 12500​ | 25​ |
| 10000​ | 48​ | 11000​ | 10​ |
| 10000​ | 72​ | 11700​ | 17​ |
| 12000​ | 0​ | 11200​ | 6.67​ |
| 12000​ | 24​ | 14800​ | 23.33​ |
| 12000​ | 48​ | 12040​ | 0.33​ |
| 12000​ | 72​ | 13100​ | 9.17​ |
| 16000​ | 0​ | 17700​ | 10.63​ |
| 16000​ | 24​ | 15900​ | 0.63​ |
| 16000​ | 48​ | 14600​ | 8.75​ |
| 16000​ | 72​ | 19100​ | 19.38​ |
| 20000​ | 0​ | 21200​ | 6​ |
| 20000 | 24​ | 17800​ | 11​ |
| 20000 | 48​ | 18500​ | 7.5​ |
| 20000 | 72​ | 20700​ | 3.5​ |


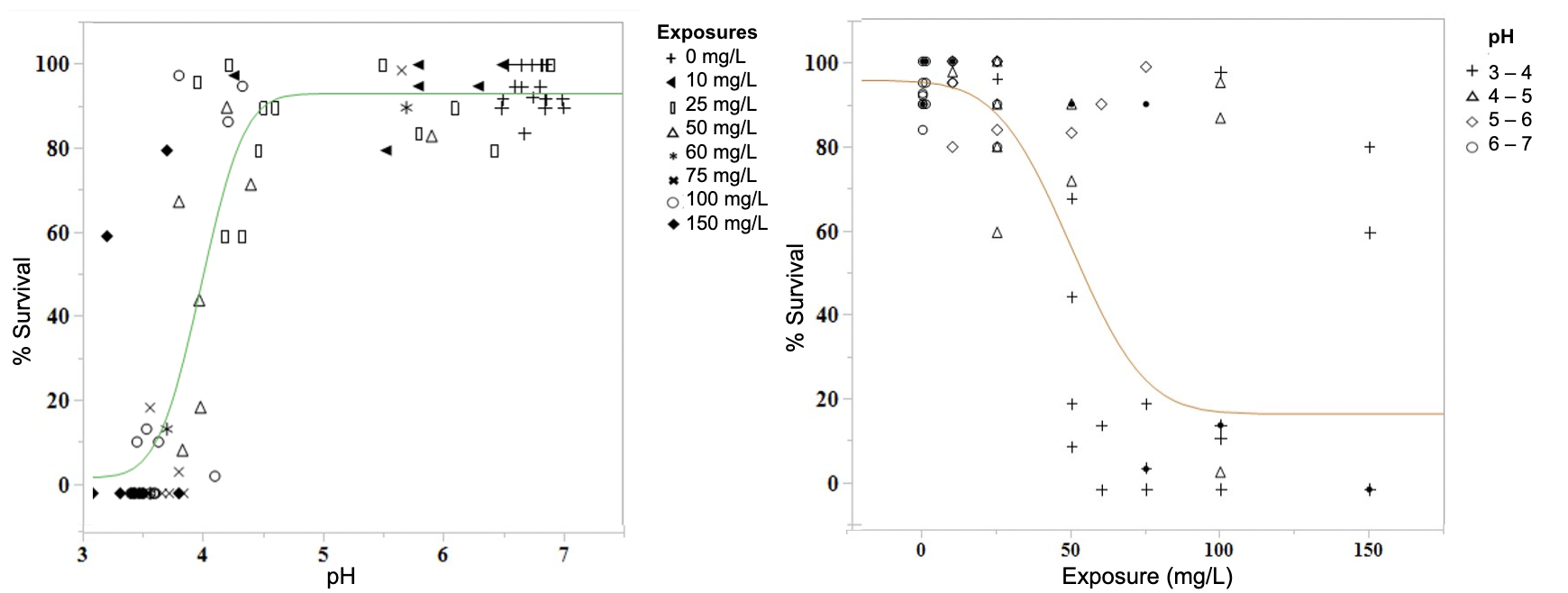


**Figure S1, Survival and pH. Correlation between percentage survival and exposure media parameters at 72 HPF.** A) Percent survival and pH of untitrated exposure media (R^2^ = 0.80). B) Percent survival and untitrated HFPO-DA concentration (R^2^ = 0.65). Survival of embryos exposed to untitrated HFPO-DA resulted in lower lethal concentrations than with titrated HFPO-DA. The correlation between pH and survival is higher than of HFPO-DA concentration and survival. Points represent trials of at least 10 embryos. Curves were fit using Probit analysis.​


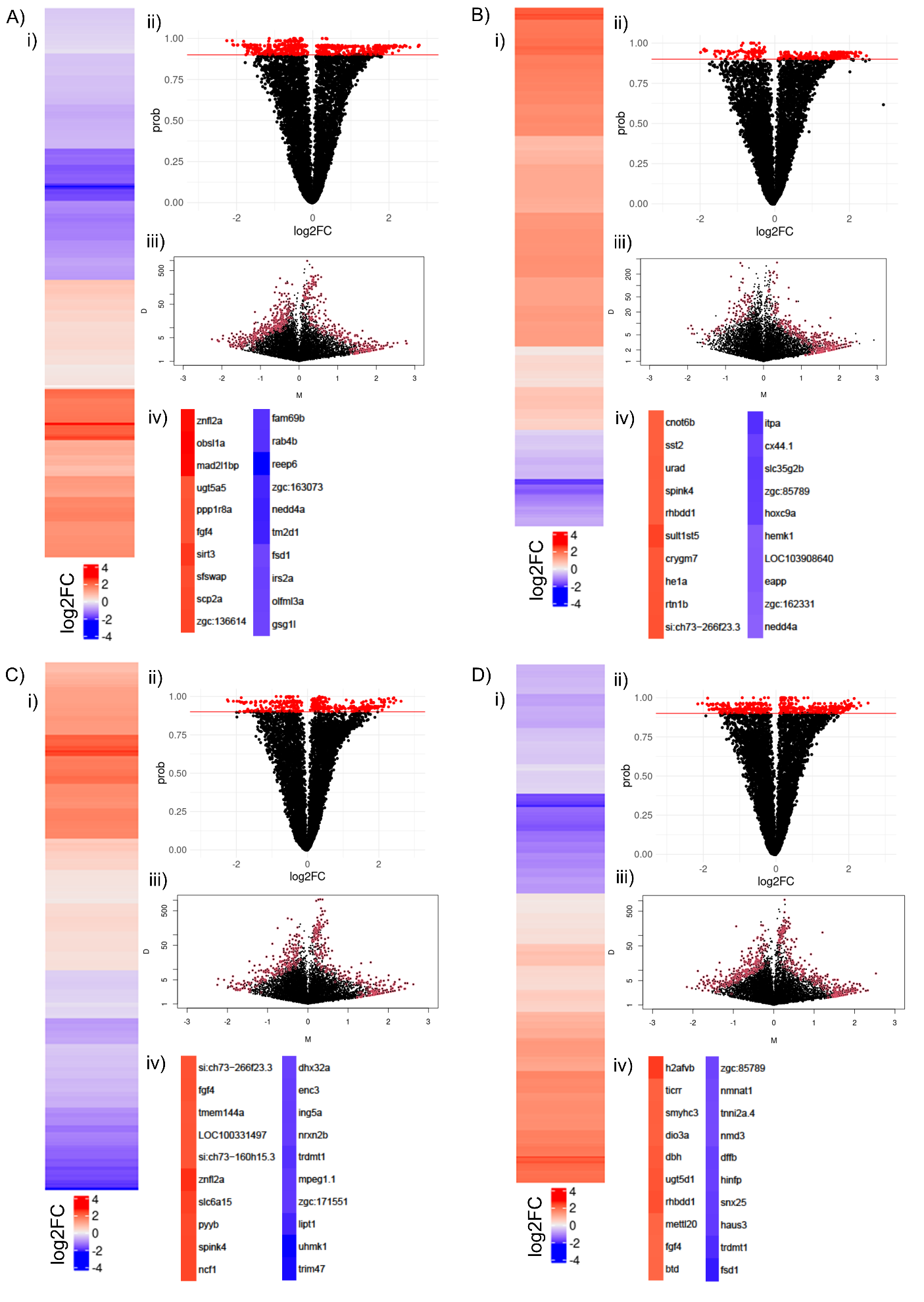


**Figure S2,** **Differentially expressed gene (DEG) expression (FDR < 0.1) between control and 0.5, 1, 2, and 10 mg/L HFPO-DA exposures on zebrafish embryos at 72 hpf.** Relative to the control condition, there were A) 616 DEGs in the 0.5 mg/L exposure, B) 487 DEGs in the 1 mg/L exposure, C) 514 DEGs in the 2 mg/L exposure, and D) 584 DEGs in the 10 mg/L. Log_2_ fold changes were calculated using 3-4 biological replicates in the NOISeq R package. Heatmaps (i) show up- (red) and down- (blue) regulation of genes within each set hierarchically clustered using Ward’s method. Each column represents an exposure, and each row represents a gene. Volcano plots (ii) show probability of differential expression (0 to 1) plotted against log_2_ fold change from -3 to 3 (0.125- to 8- fold change). Red points have probabilities higher than a threshold of 0.9 (indicated by red line) and are significant. Mean-difference (MD) plots (iii) show absolute difference of expression (D) plotted against log_2_ fold change (M). The top and bottom 10 expressed genes are listed in (iv) for each exposure.


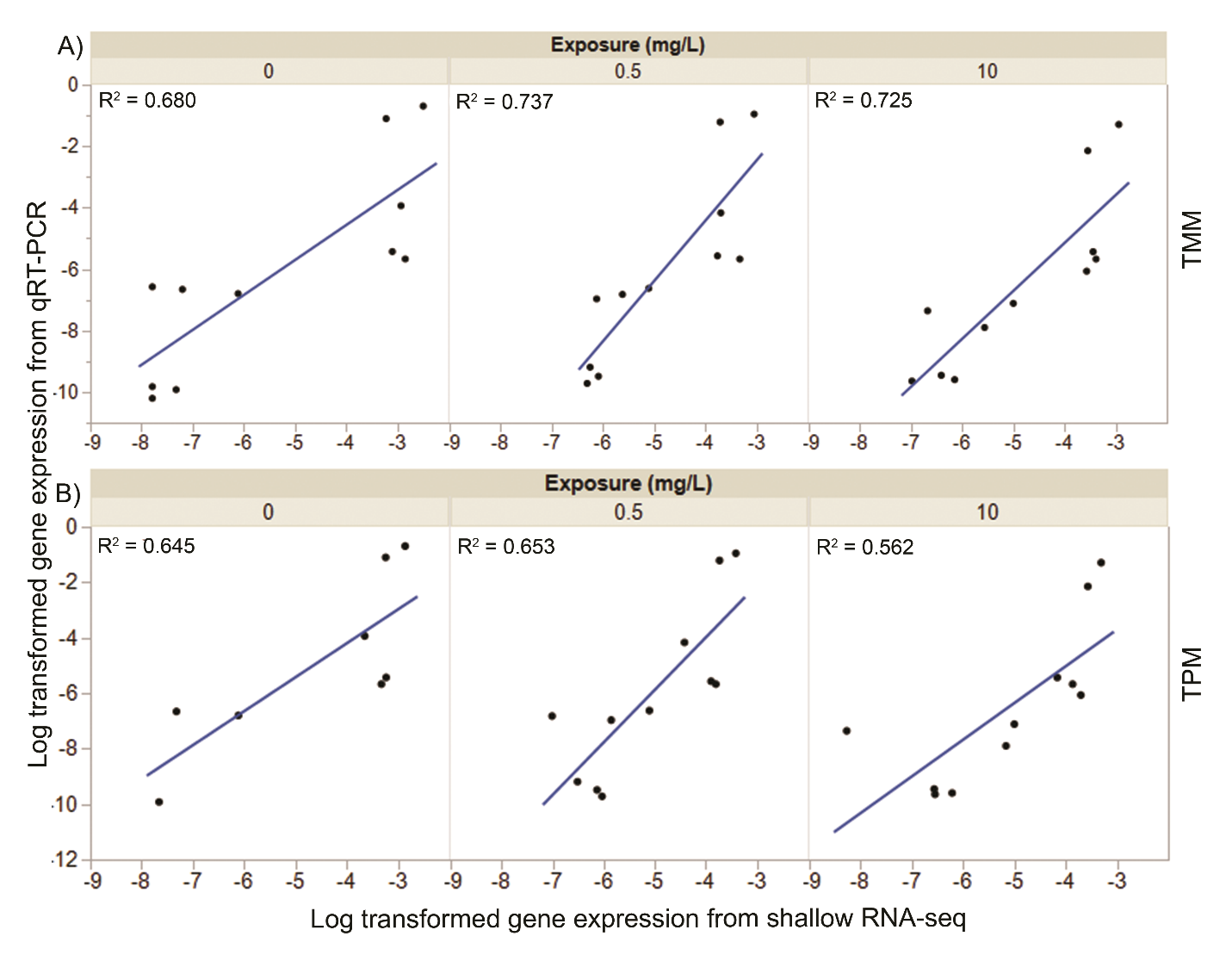


**Figure S3. Correlations of qRT-PCR and shallow RNA-Seq gene expression normalized to housekeeping gene.** Y-values represent log_e_-transformed gene expression calculated from ∆Cts in qRT-PCR. X-values represent log_e_-transformed A) TMM- and B) TPM-normalized, then housekeeping gene *actb1*-normalized gene expression from shallow RNA-Seq results. Genes were selected from highly upregulated (6) and downregulated (5) differentially expressed genes (relative to control) based on shallow RNA-Seq.
